# Do wastewater treatment plants increase antibiotic resistant bacteria or genes in the environment? Protocol for a systematic review

**DOI:** 10.1186/s13643-019-1236-9

**Published:** 2019-12-05

**Authors:** Daloha Rodríguez-Molina, Petra Mang, Heike Schmitt, Mariana Carmen Chifiriuc, Katja Radon, Laura Wengenroth

**Affiliations:** 1 Occupational and Environmental Epidemiology & NetTeaching Unit, Institute and Clinic for Occupational, Social and Environmental Medicine, University Hospital, LMU Munich, Ziemssenstr. 1, 80336 Munich, Germany; 2 Centre of Infectious Disease Control, National Institute for Public Health and the Environment, Antonie van Leeuwenhoeklaan 9, 3721 MA Bilthoven, The Netherlands; 3 Department of Microbiology and Immunology, Faculty of Biology, University of Bucharest, Bucharest, Romania; 4 Earth, Environmental and Life Sciences Section, Research Institute of the University of Bucharest, University of Bucharest, Bucharest, Romania

**Keywords:** Antibiotic resistance bacteria, Antibiotic resistance genes, Wastewater treatment plants, Environmental samples, Systematic review protocol

## Abstract

**Background:**

Antibiotic resistance is a global public health threat. Water from human activities is collected at wastewater treatment plants where processes often do not sufficiently neutralize antibiotic resistant bacteria and genes, which are further shed into the local environment. This protocol outlines the steps to conduct a systematic review based on the Population, Exposure, Comparator and Outcome (PECO) framework, aiming at answering the question “Are antimicrobial-resistant enterobacteriaceae and antimicrobial resistance genes present (O) in air and water samples (P) taken either near or downstream or downwind or down-gradient from wastewater treatment plants (E), as compared to air and water samples taken either further away or upstream or upwind or up-gradient from such wastewater treatment plant (C)?” Presence of antimicrobial-resistant bacteria and genes will be quantitatively measured by extracting their prevalence or concentration, depending on the reviewed study.

**Methods:**

We will search PubMed, EMBASE, the Cochrane database and Web of Science for original articles published from 1 Jan 2000 to 3 Sep 2018 with language restriction. Articles will undergo a relevance and a design screening process. Data from eligible articles will be extracted by two independent reviewers. Further, we will perform a risk of bias assessment using a decision matrix. We will synthesize and present results in narrative and tabular form and will perform a meta-analysis if heterogeneity of results allows it.

**Discussion:**

Antibiotic resistance in environmental samples around wastewater treatment plants may pose a risk of exposure to workers and nearby residents. Results from the systematic review outlined in this protocol will allow to estimate the extend of exposure, to inform policy making and help to design future studies.

## Background

Antibiotic resistance has become an imminent global public health threat and multiple studies have identified resistant bacteria and resistance genes in environmental samples [1, 2]. Water resulting from human activities such as agriculture, healthcare services and from the general population is collected at wastewater treatment plants (WWTPs), turning them into unintentional collection points for antimicrobial drugs, antibiotic-resistant bacteria (ARB) and resistance genes (ARG) [3]. Wastewater treatment processes are not designed to remove ARB and ARG, so WWTPs typically harbor antimicrobials and other agents known to co-select for antibiotic resistance [4, 5]. These ARB and ARG present in the air and water in and around WWTP may spread depending on proximity to workers and nearby residents, and they are shed into outgoing environmental systems such as rivers and reservoirs [1].

This protocol describes the methodology that we will use to evaluate presence of ARB and ARG from *E. coli* and other enterobacteriaceae in air and water samples from WWTPs and to find out especially if these levels are higher in close proximity to WWTPs. To reduce a potential high level of heterogeneity regarding the setting, the type of samples, and the species and strains of bacteria and genes [6, 7], our systematic review will focus specifically on answering the following research question: Are antimicrobial-resistant enterobacteriaceae and antimicrobial resistance genes present (O) in air and water samples (P) taken either near or downstream or downwind or down-gradient from a wastewater treatment plant (E), as compared to air and water samples taken either further away or upstream or upwind or up-gradient from such wastewater treatment plant (C)? Presence of antimicrobial-resistant bacteria and genes will be quantitatively measured by extracting their prevalence or concentration, depending on the reviewed study.

## Review team roles and responsibilities

Information about the team members working in this systematic review along with their applicable knowledge and skills and their responsibilities can be seen in Table 1.

**Table 1:** Team members of the planned systematic review

Team member	Applicable knowledge and skills	Responsibilities
Daloha Rodriguez-Molina	Epidemiologic methods, antibiotic resistance epidemiology, microbiology, systematic review and meta-analysis methods, clinical experience with antibiotics, occupational and environmental epidemiology	Create and describe literature research method, study screening, data extraction, evidence evaluation, content drafting and approval, statistical analysis (if applicable)
Petra Mang	Microbiology and epidemiology of antibiotic resistance, systematic review methods, clinical experience with antibiotics	Study screening, data extraction, evidence evaluation
Heike Schmitt	Microbiology of antibiotic resistance in the environment, veterinary medicine, transmission of resistant bacteria with surface water and manure, WWTP expertise, human exposure to antimicrobial resistant factors and carriage of exposed populations, systematic review methods.	Systematic review methods consultation, content review and approval
Mariana Carmen Chifiriuc	Microbiology of antibiotic resistant bacteria and resistance genes in the environment, human exposure to antimicrobial resistant factors and carriage of exposed populations.	Microbiology consultation, content review and approval
Laura Wengenroth	Epidemiologic methods, antibiotic resistance epidemiology, systematic review and meta-analysis methods, social sciences, occupational and environmental epidemiology	Systematic review methods consultation, content review and approval
Katja Radon	Epidemiologic methods, systematic review and meta-analysis methods, environmental engineering, occupational and environmental epidemiology	Systematic review methods consultation, content review and approval

## Methods

### Eligibility criteria

**Fig. 1:**
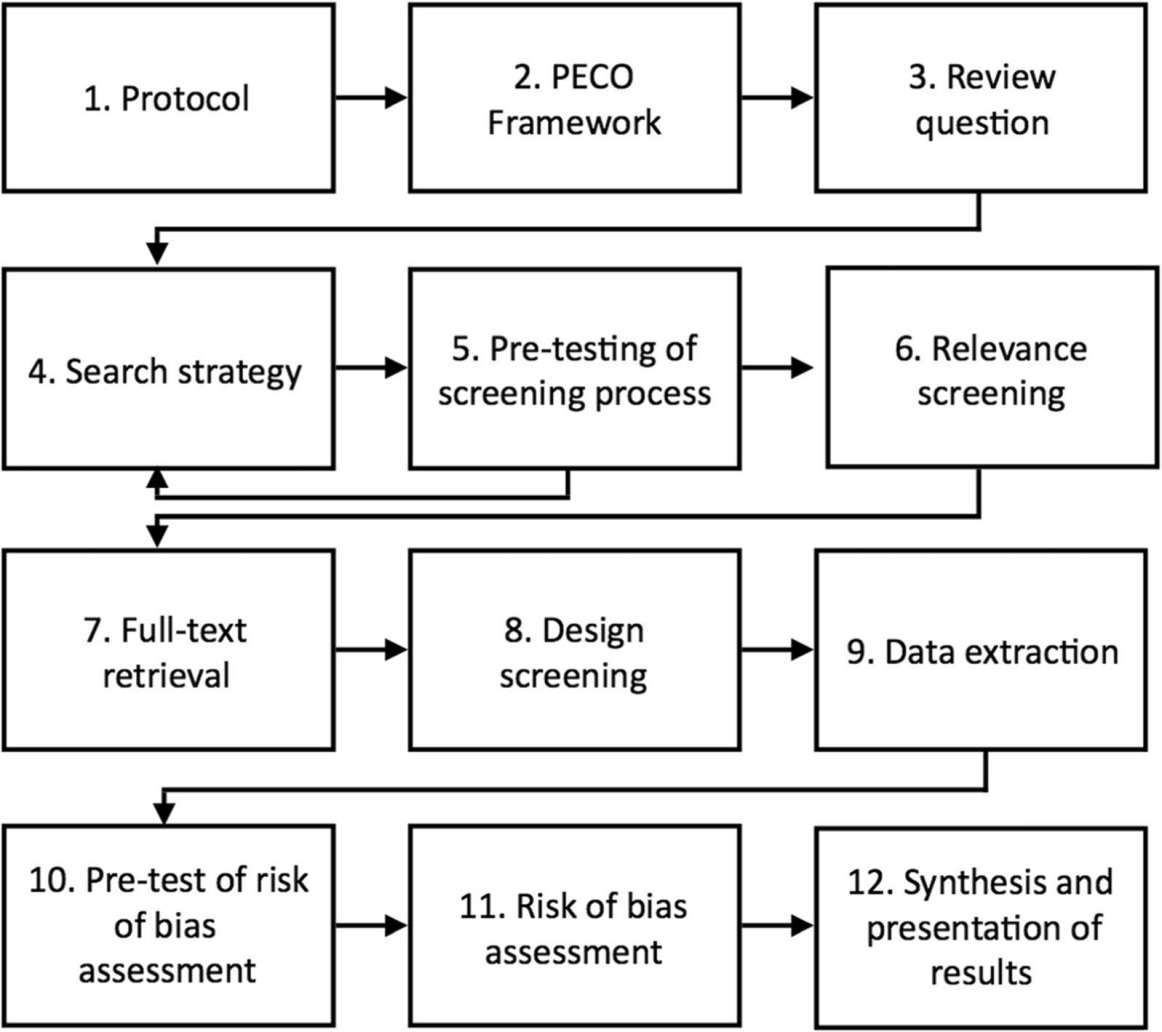
Steps of the systematic review process

This systematic review will be conducted following the general steps outlined in Fig. 1, from writing the protocol to extracting the data. We have constructed our research question following the PECO framework (Table 2). Our *P*opulation of interest is air and water samples from WWTPs; our *E*xposure is locations near WWTPs, or downstream/downwind/down-gradient from the WWTP in a unidirectional system. Our *C*omparison group refers to locations far away from the WWTPs, or upstream/upwind/up-gradient from the WWTP in a unidirectional system. Our *O*utcome is the presence of antimicrobial-resistant bacteria, especially *E. coli* and other coliforms, and their resistance genes, measured by extracting either their prevalence or concentration in the reported samples, depending on the reviewed study. We expect to retrieve mostly cross-sectional studies.

**Table 2:** Eligibility criteria using the PECO framework

*P*opulation	Air and water samples from WWTPs
*E*xposure	Locations near WWTPs, or downstream/downwind/down-gradient from this plant in a unidirectional system
*C*omparator	Locations far away from WWTPs, or upstream/upwind/up-gradient from this plant in a unidirectional system
*O*utcome	Prevalence or concentration of ARB or ARG, specifically from *E. coli* and other coliforms

*ARB* antibiotic-resistant bacteria, *ARG* antibiotic resistance genes, *WWTPs* wastewater treatment plants

Although we hypothesize that the closer to a WWTP, the higher the quantitative presence of ARB and ARG, a cut-off point for this distance has not been established for anthropogenic sources [8]. Therefore, “proximity”, “close distance” and other similar terms will not be defined for the purposes of this systematic review.

### Sources of information

We will perform a thorough and rigorous search of the current literature using several electronic databases, specifically PubMed, EMBASE, the Cochrane database and Web of Science, including its sub-databases such as Biosis Citation Index, Core Collection, Current Contents Connect and Scielo. After having performed preliminary searches, we have realized that before the year 2000 studies about antimicrobial resistance were mostly focused on the clinical setting and not on environmental samples. We have therefore decided to look for all scientific articles with full-texts published in English, German, Spanish, Italian, Portuguese and French, from January 1, 2000 to September 3, 2018.

We will not perform hand-search of journals, conference proceedings, theses or dissertations, nor will we use web search engines (e.g. Google Scholar) as these methods are typically not precise enough, difficult to reproduce and time consuming [9, 10].

### Search strategy

Our search strategy for PubMed is the following:

((”drug resistance, microbial“[Mesh] AND”Escherichia coli“[Mesh:NoExp] OR”Carbapenem-Resistant Enterobacteriaceae“[Mesh] OR”Enterobacter“[MAJR]) AND (”Water Microbiology“[Mesh] OR ”Waste Water/microbiology“[MAJR] OR ”Air Microbiology“[Mesh]) AND (”humans“[MeSH Terms] OR ”waste water“[MeSH Terms] OR ”sewage“[MeSH Terms]) NOT Review[Publication Type])

Similar search strategies adapted to other databases will be generated after this one and using the Systematic Review Accelerator web tool [11].

### Data management

Titles and abstracts of identified articles will be imported into EndNote X8.2 (Thomson Reuters). The screening process will be done using Rayyan QCRI, a web application to review articles for a systematic review [12]. Screening, risk of bias assessment and data extraction will be performed with the help of forms and relational databases in Access 2016 (Microsoft Corporation, Redmond, WA, USA). Statistical analyses, if needed for a meta-analysis of the reviewed literature, will be performed using R for mac version 3.5.0 or higher [13].

### Study selection

#### Relevance screening

The first stage in our systematic review will be the relevance screening. The main objective of this stage is to check if articles comply with the research question and are eligible for further review. Two reviewers will independently assess titles and abstracts of all retrieved articles. Consensus between reviewers will be required and potential conflicts will be resolved in meetings every two weeks during the relevance screening phase. If it is not possible to achieve consensus between the two assigned reviewers, a third reviewer from the team will be consulted as a tie-breaker. This stage will be carried out with the help of a standardized questionnaire based on previous works [6, 7, 14] and refined for the purposes of our review. This instrument and screening methods will be pre-tested by taking a random sample of *n* = 30 retrieved abstracts. The main aim of the pre-test is to make sure that there are no misunderstandings from the reviewers regarding the use of the screening tool or the user interface of the software used to track screening.

Our tool includes the following questions:

1. Does the abstract refer to primary research reported in a journal publication or a thesis (as opposed to a review article or presentation abstract or proceedings)?
2. Were samples of water or air collected from the exterior environment (i.e. not in a building or internal facility)?
3. Were the samples collected from a WWTP as point source?
4. Does the study measure either the prevalence or the concentration of antibiotic resistance factors (bacteria or genes) for enterobacteria in the samples?
5. Does the study use microbial source tracking techniques?

Possible answers to each one of these questions are “Yes”, “No” and “It is not clear from the title and abstract.” Based on our research question, we will consider articles if they report findings from primary research, collected environmental samples of air and water from a WWTP and report the prevalence or concentration of antibiotic resistance factors -either bacteria or genes- from *Enterobacteriaceae*. Further, studies will be excluded if they used microbial source tracking techniques because these techniques aim at identifying the source of bacterial isolates or strains in environmental samples and not at comparing the concentration or proportion of resistant isolates in different locations [6, 7]. In other words, studies will be included in our systematic review if the answer to questions 1 to 4 is “yes” and to question 5 is “no”, and will be excluded if the answer to either of the questions 1 through 4 is “no” or the answer to question 5 is “yes”. If the title and abstract of the article do not allow us to reach a clear yes/no answer to these questions, we will classify the article as “unclear” and it will remain available for discussion and further clarification.

#### Design screening

The next stage is the design screening. The main objective of this stage is to check whether articles considered proximity to a WWTP as an exposure variable, either by comparing proximity to one or more comparison groups or to a range of distances. We will also consider direction from the WWTP in a unidirectional system, e.g. comparing downstream vs. upstream water samples in a river system adjacent to a WWTP. We will additionally check if the article is available in any of the following languages: English, German, Spanish, Italian, Portuguese or French.

In order to achieve the main aim of this stage, we will retrieve the full-text document of publications remaining after the relevance screening stage and read only the methods section, omitting the results and conclusions sections. We will use a standardized design screening questionnaire containing the following questions:

6. Does the study implicitly or explicitly define a WWTP as a point source with reference to which a comparison was defined?
7. Does the study report *proximity to* or *direction from* a WWTP?
8. Does the study specify a comparison group for the samples? (e.g. samples taken upstream from the source or at a fixed distance from the source)
9. Is the article written in English, German, Spanish, Italian, Portuguese or French?

Articles will remain in our systematic review for further data extraction and analysis if the answer to each of the previous questions is “yes”. If the answer to any of the questions is “no”, the article will be excluded without further consideration. Decision conflicts and articles to which the answer to any of the questions 1 to 3 is “unclear” will be discussed by the two reviewers and inclusion will be decided on a case-by-case basis. Articles that meet inclusion criteria after relevance and design screening will be considered well-suited to answer our research question and will continue onto the next stage, where we will extract the data and appraise the quality of each study.

### Data extraction

Extracting relevant data from each study will facilitate evidence synthesis, interpretation and presentation of results. Data will be independently extracted by the two reviewers and inputted into a relational database structured form that will be pre-piloted with a sample containing 10% of the citations eligible at this stage. A third reviewer will be consulted for arbitration in case of disagreements.

We will focus on these main categories for data extraction: (a) type of sample, source and outcome, (b) sample size, (c) statistical parameters in case modelling was used, (d) confounding control and (e) measure of resistance. Details on the specific values to extract for each of these categories are given in Table 3.

**Table 3:** Data to be extracted from each study

Category	Parameter to extract
Descriptive characteristics of the study	• Location of the study
	• Study design
Type of sample, source and outcome	• Environmental media or biological sample type tested
	• Location of the WWTP where the sample(s) was (were) taken
	• Quantity of samples
	• Bacterial species and/or genes analyzed
	• Microbiological method used
Type of exposure	• Categorical: Downstream, downwind or down-gradient
	• Continuous: Distance to the WWTP; distance to the Comparator
Type of comparator	• Categorical: Upstream, upwind or up-gradient
	• Continuous: Distance to the WWTP; distance to the exposure site?
Sample size	• If exposure is categorical: Sample size of each group
	• If exposure is continuous: Total sample size
If statistical modelling was used	• Type of model
	• Effect measure (extracted or computed if there is enough raw data reported)
	• Measure of variability
	• *p* value
Confounding control	• Methods to account for confounding or account for clustering data
Measure of resistance	• Proportional: Is denominator present? Was the denominator stable across sampling sites?
	• Absolute

### Risk of bias assessment

We will identify threats to internal validity in each of the remaining articles by performing a risk of bias assessment based on previously published methods [14]. Because we are aiming at identifying threats to internal validity, and not precision, quality or external validity, we will not consider aspects such as statistical power, representativeness, or adequate choice of statistical methods. Statistical power is more related to precision than to external validity [15], representativeness refers to external validity, and adequate choice of statistical methods is a measure of quality. For the purposes of our review, we define quality as the best that the researchers could have done under the specific circumstances of their study, and not as whether there is a threat to internal validity. For example, in a randomized controlled trial of surgery vs. a pharmaceutical gold standard for a given health-related outcome, it is impossible to blind researchers or participants. This study should still be considered of high quality if the proposing treatment (surgery) is the best candidate against the established gold standard. However, this study will inevitable be at risk of bias because it was not blinded.

For each publication, our risk of bias assessment will be performed at the study design level and not at the outcome level because we are interested in evaluating if the techniques used in the included studies were sufficient to avoid potential bias or if, on the contrary, bias could have been introduced by design. At least two reviewers will evaluate the risk of bias at each of these domain levels: sample selection bias, information bias, and confounding. Following an adaptation from the Cochrane Collaboration Risk of Bias Tool [15], we will classify risk of bias to each domain as low, high or unclear. Further, we will determine the overall risk of bias for each study as follows: if there is a low risk of bias in all domains, the overall risk of bias will be low. If the risk of bias is unclear in at least one of the three domains, the overall risk of bias will be classified as unclear. Similarly, if the risk of bias for one or more domains is high, the overall risk of bias will be considered high. An overall low risk of bias means that plausible bias is not likely to change results, while an overall high risk of bias means that plausible bias may risk confidence in results. When the overall risk of bias is unclear, it is accepted that there are some doubts about the results.

Assessing risk of bias in each domain follows answering a specific question yes/no for each domain when judging different study aspects or methodologies with specific criteria. Criteria for the judgement of “yes” translate into low risk of bias, and vice versa with criteria for the judgement of “no”. Criteria will be judged as “unclear” if there is not enough information to reach a yes/no answer. The specific question to be answered at each domain level along with criteria for the judgement of yes, no or unclear is shown in Table 4.

**Table 4:** Risk of bias assessment

Bias domain	Assessment question	Criteria
Sample selection bias	Were *sample locations and sampling methods* implemented such that sampling did not introduce systematic differences depending on the value of the exposure variable for each sample (in the case of continuous exposure data) or between the comparison groups (in the case of categorical exposure measures)?	1. Criteria for the judgement of “Yes”: • Method for determining the sampling locations is identical independent of exposure status (i.e. distance or direction from source); • Restriction of sampling locations is applied in the same way regardless of exposure status (e.g. sampling sites are all agricultural fields with a similar type and level of historical use); • Time between sampling at all sites is sufficiently close so as to render the outcomes measured at these sites comparable for the sample type in question
		2. Criteria for the judgement of “No”: • Sampling locations are selected differently; • Restriction of sample locations is applied differently depending on exposure status
		3. Risk of bias will be considered “unclear” if there is not enough information to judge sample selection bias criteria as either “yes” or “no”, e.g. if methods for determining sampling locations are not described in enough detail
Information bias	“Were *outcome ascertainment methods* (i.e. methods of gene or bacterial measurement) conducted in a way that ensures the same accuracy regardless of distance or direction from the source(s)?”	1. Criteria for the judgement of “Yes”: • Identical microbiological methods applied to all samples; • Controlling for laboratory factors, if these are different (e.g. which laboratory, technician, testing date, instrument used); • Blinding laboratory staff to exposure status
		2. Criteria for the judgement of “No”: • Application of different methods depending on comparison group; • No adjustment strategy for different laboratory methods
		3. Risk of bias will be considered “unclear” if there is not enough information to judge information bias criteria as either “yes” or “no”, e.g. if methods for analyses are not explained sufficiently to reach a judgement
Confounding^a^	“Were adequate methods to *control for potential confounding* employed?”	1. Criteria for the judgement of “Yes”: • Restriction of the sample population; • Analytical confounding control (e.g. stratification, regression adjustment)
		2. Criteria for the judgement of “No”: • Lack of any confounding control despite confounding being likely; • Inappropriate method of confounding control used; • Controlling for confounding is correctly applied for some potential confounders, but not for all
		3. Risk of bias will be considered “unclear” if there is not enough information to judge information bias criteria as either “yes” or “no”, e.g. if methods to control for confounding are mentioned but the implementation is not explained sufficiently at length to reach a judgement

^a^Some potential confounders for measuring antibiotic resistant bacteria and genes in environmental samples such as air and water samples include varying bacterial population size across sampling locations, environmental media composition (e.g. water salinity), recent precipitation and other weather events, sample composition and other sources of antibiotics or antibiotic resistance factors [18]

The risk of bias assessment procedure will be pre-tested using a random sample of 10 % of the retrieved articles until this point. The main aim of the pre-test is to make sure that there are no misunderstandings from the reviewers regarding the risk of bias assessment criteria or the relational databases used to track this stage.

### Evidence synthesis

We will report our results narratively and in tabular form, presenting the information extracted from each of the articles included in our review and our risk of bias assessment. We will provide descriptive characteristics of each of the studies (year, country, WWTP characteristics if available, type of sample collected) and of their outcomes (examined bacteria or genes, relevant comparison methods, relevant findings including effect measures), as well as our judgement for the risk of bias assessment and the reason(s) for that given judgement. If there is an enough number of high-quality studies with a relatively low level of heterogeneity among effect measures, we will pool findings into a fixed-effects or random-effects meta-analysis and report these results using forest and funnel plots.

## Discussion

In this protocol, we have described our planned methodology to conduct a rigorous revision and synthesis of the published literature regarding the prevalence or concentration of antibiotic-resistant enterobacteria and resistance genes in air and water samples around WWTPs. Our pre-specified criteria for searching the literature, screening relevant articles, assessing risk of bias and extracting and presenting findings will guide us through all of the steps of our review and help us avoid introducing bias *a posteriori*.

We can however anticipate some *a priori* sources of bias for our review. Some studies only appear in conference proceedings and do not reach a journal publication stage. Excluding conference proceedings puts our review at risk of containing publication bias. However, conference proceedings usually show only a short abstract of each study, which does not allow proper screening or assessment, making these procedures time-consuming and often leading to fruitless results [10]. Further, hand-searching of journals, conference proceedings and the use of web search engines (e.g. Google scholar) is laborious, time-consuming, and seldom improve the quality of systematic reviews [9]. We expect our methods to ensure reproducibility while maintaining appropriate levels of sensitivity and specificity. Another foreseeable source of bias comes from our language restriction, which may introduce bias and lower precision. However, previous meta-analyses on the impact of language bias in systematic reviews and meta-analyses of controlled trials have found that excluding trials published in languages other than English has a little effect on summary treatment effect estimates [16, 17]. Therefore, by including English and other Indo-European languages as a criterion for inclusion, we minimize introducing language bias. Another strength of our methods is that, in order to ensure a transparent and complete reporting, we will follow the Preferred Reporting Items for Systematic Reviews and Meta-Analysis (PRISMA) [18]. A complete PRISMA checklist for this protocol can be found in the Additional file 1.

To our knowledge, there are at least two previously published systematic reviews with similar questions to ours [6, 7]. These highly informative works present a high level of study heterogeneity that prevented pooling results. We believe that our systematic review will give an update and will allow to draw more precise results focusing only on antibiotic-resistant enterobacteria and resistance genes in air and water samples around WWTPs. Further, we aim at widening our search of this more specific research question by using a different set of databases, including EMBASE, the Cochrane database, Web of Science and its sub-databases, thus providing a broader understanding of the research question focused on antibiotic-resistant enterobacteria and resistance genes in air and water samples around WWTPs.

Although our planned systematic review is in the field of environmental sciences, it is highly related to health-related outcomes. Antibiotic-resistant enterobacteria and resistance genes found in environmental samples around WWTPs may pose a risk to the health status and well-being of WWTP workers and nearby residents. Quantifying the prevalence or concentration of antibiotic-resistant bacteria and genes in anthropogenic sources will improve our understanding of the magnitude of the risk to which humans are exposed. In addition, our study results will provide guidance and support for planning future studies and in the end to establish further requirements for the usage of antimicrobial drugs, and for the treatment of wastewater polluted with antibiotic-resistant bacteria and resistance genes.

## Supplementary information

**Additional file 1.** PRISMA-P 2015 Checklist.

## Data Availability

Not applicable.

## Abbreviations

ARB: Antibiotic-resistant bacteria
ARG: Antibiotic-resistant genes
PECO: Population, Exposure, Comparator and Outcome framework
WWTP: Wastewater treatment plants
