# Supplementary material for: Do wastewater treatment plants increase antibiotic resistant bacteria or genes in the environment? Protocol for a systematic review
Source: Syst Rev. 2019 Dec 5;8:304. doi: 10.1186/s13643-019-1236-9 (PMC6894476; doi:10.1186/s13643-019-1236-9)
Supplement: Supplementary file 1 — Additional file 1. PRISMA-P 2015 Checklist. [file 13643_2019_1236_MOESM1_ESM.pdf]

Rodriguez Molina, Daloha Virginia

**Von:**	CRD-REGISTER <irss505@york.ac.uk>
**Gesendet:**	Mittwoch, 8. Mai 2019 16:10
**An:**	Rodriguez Molina, Daloha Virginia
**Betreff:**	PROSPERO Registration message [127357]

Dear Ms Rodriguez-Molina,

Thank you for submitting details of your systematic review "Do wastewater treatment plants increase antibiotic resistant bacteria or genes in the environment? Protocol for a systematic review" to the PROSPERO register.

We regret that, based on the information you have provided, your review falls out of the scope of PROSPERO. Systematic reviews of in-vitro studies are not eligible for inclusion in PROSPERO and cannot be registered.

Once rejected the record cannot be further amended and access to the record is not possible without contacting us by email at crd-register@york.ac.uk

You may use this notification to advise funders or journals that it is not possible to register studies that are not done in humans in PROSPERO.

We hope that this will not discourage you from registering future eligible systematic reviews with PROSPERO.

Yours sincerely,

PROSPERO Administrator
Centre for Reviews and Dissemination
University of York
York YO10 5DD
t: +44 (0) 1904 321049
e: CRD-register@york.ac.uk
www.york.ac.uk/inst/crd

PROSPERO is funded by the National Institute for Health Research and produced by CRD, which is an academic department of the University of York.

Email disclaimer: https://www.york.ac.uk/docs/disclaimer/email.htm
